# Optimization of Nutrition And Medication (OptiNAM) for acutely admitted older patients: protocol for a randomized single-blinded controlled trial

**DOI:** 10.1186/s13063-021-05456-6

**Published:** 2021-09-14

**Authors:** Aino L. Andersen, Morten B. Houlind, Rikke L. Nielsen, Lillian M. Jørgensen, Charlotte Treldal, Morten Damgaard, Anne Kathrine Bengaard, Helle Gybel Juul-Larsen, Louise Bolvig Laursen, Esben Iversen, Marie Kruse, Anne M. L. Pedersen, Mads Hornum, Anne M. Beck, Mette M. Pedersen, Mikkel Z. Ankarfeldt, Janne Petersen, Ove Andersen

**Affiliations:** 1 Department of Clinical Research, Copenhagen University Hospital Amager and Hvidovre, Kettegaards alle 30, 2650 Hvidovre, Denmark; 2 Department of Clinical Medicine, Faculty of Health and Medical Sciences, University of Copenhagen, Blegdamsvej 3B, 2200 Copenhagen N, Denmark; 3 The Capital Region Pharmacy, Marielundsvej 25, 2730 Herlev, Denmark; 4 Department of Drug Design and Pharmacology, University of Copenhagen, Universitetsparken 2, 2100 Copenhagen Ø, Denmark; 5 Emergency Department, Copenhagen University Hospital Amager and Hvidovre, Kettegaards alle 30, 2650 Hvidovre, Denmark; 6 Department of Clinical Physiology and Nuclear Medicine, Centre for Functional and Diagnostic Imaging and Research, Copenhagen University Hospital Amager and Hvidovre, Kettegaards alle 30, 2650 Hvidovre, Denmark; 7 Department of Physio- and Occupational Therapy, Copenhagen University Hospital Amager and Hvidovre, Kettegaards alle 30, 2650 Hvidovre, Denmark; 8 Danish Centre for Health Economics, University of Southern Denmark, J.B. Winsløws Vej 9B, 5000 Odense C, Denmark; 9 Section of Oral Medicine and Pathology, Department of Odontology, Faculty of Health and Medical Sciences, University of Copenhagen, Copenhagen N, Denmark; 10 Department of Nephrology, Copenhagen University Hospital Rigshospitalet, Inge Lehmanns Vej 7, Copenhagen Ø, Denmark; 11 Department of Nursing and Nutrition, University College Copenhagen, Sigurdsgade 26, 2200 Copenhagen N, Denmark; 12 Dietetic and Nutritional Research Unit, Herlev-Gentofte University Hospital, Borgmester Ib Juuls Vej 50, 2730 Herlev, Denmark; 13 Copenhagen Phase IV unit (Phase4CPH), Center of Clinical Research and Prevention and Department of Clinical Pharmacology, Copenhagen University Hospital Bispebjerg and Frederiksberg, Nordre Fasanvej 57, 2000 Frederiksberg, Denmark; 14 Section of Biostatistics, Department of Public Health, University of Copenhagen, Øster Farimagsgade 5, 1014 Copenhagen K, Denmark

**Keywords:** Drug utilization Review, Potentially inappropriate medication list, Glomerular filtration rate, Pharmacogenetics, Malnutrition, Quality of life, Gastrointestinal microbiome, Frailty, Geriatrics, Emergency service, Hospital

## Abstract

**Background:**

Internationally, older patients (≥65 years) account for more than 40% of acute admissions. Older patients admitted to the emergency department (ED) are frequently malnourished and exposed to inappropriate medication prescribing, due in part to the inaccuracy of creatinine-based equations for estimated glomerular filtration rate (eGFR). The overall aims of this trial are to investigate: (1) the efficacy of a medication review (MED intervention) independent of nutritional status, (2) the accuracy of eGFR equations based on various biomarkers compared to measured GFR (mGFR) based on ^99m^Technetium–diethylenetriaminepentaacetic acid plasma clearance, and (3) the efficacy of an individualized multimodal and transitional nutritional intervention (MULTI-NUT-MED intervention) in older patients with or at risk of malnutrition in the ED.

**Methods:**

The trial is a single-center block randomized, controlled, observer-blinded, superiority and explorative trial with two parallel groups. The population consists of 200 older patients admitted to the ED: 70 patients without malnutrition or risk of malnutrition and 130 patients with or at risk of malnutrition defined as a Mini Nutritional Assessment-Short Form score ≤11. All patients without the risk of malnutrition receive the MED intervention, which consists of a medication review by a pharmacist and geriatrician in the ED. Patients with or at risk of malnutrition receive the MULTI-NUT-MED intervention, which consists of the MED intervention in addition to, dietary counseling and individualized interventions based on the results of screening tests for dysphagia, problems with activities of daily living, low muscle strength in the lower extremities, depression, and problems with oral health. Baseline data are collected upon study inclusion, and follow-up data are collected at 8 and 16 weeks after discharge. The primary outcomes are (1) change in medication appropriateness index (MAI) score from baseline to 8 weeks after discharge, (2) accuracy of different eGFR equations compared to mGFR, and (3) change in health-related quality of life (measured with EuroQol-5D-5L) from baseline to 16 weeks after discharge.

**Discussion:**

The trial will provide new information on strategies to optimize the treatment of malnutrition and inappropriate medication prescribing among older patients admitted to the ED.

**Trail registration:**

ClinicalTrials.gov NTC03741283. Retrospectively registered on 14 November 2018.

**Supplementary Information:**

The online version contains supplementary material available at 10.1186/s13063-021-05456-6.

## Introduction

### Background

Internationally, older patients (≥65 years) account for 41–46% of acute admissions [1–3]. Among older patients admitted to the emergency department, the prevalence of the risk of malnutrition and malnutrition ranges between 35 and 71% in acutely admitted older patients [4–8] and the prevalence of polypharmacy (≥5 prescribed medications) is 73–77%, while 51–85% receive at least one potentially inappropriate medication (PIM) [9–12]. Both malnutrition and polypharmacy are associated with decreased quality of life [13–16], and these risk factors are often comorbid, as 64–87% of older patients at risk of malnutrition present with polypharmacy [5, 6, 9]. However, interventions to address malnutrition and inappropriate medication prescribing in the ED are limited by the acute setting. The median length of stay (LOS) for acutely admitted older medical patients is 2.2–4.5 days [17, 18], and more than half of all patients admitted to the ED are discharged without being transferred to another department [6, 8, 19]. Consequently, some of these patients will be discharged before initiatives to overcome malnutrition or inappropriate medication have been started. Therefore, it is essential that interventions to optimize nutritional care and medication prescribing are started in the ED and continued after discharge (transitional care).

#### Inappropriate prescribing and medication review intervention

Polypharmacy is common among older patients in part because medications are readily prescribed for new conditions, but the appropriateness of continuing each medication is rarely evaluated [20–22]. Polypharmacy and other causes of inappropriate medication prescribing may lead to adverse drug reactions, which are responsible for up to 15% of unplanned hospitalizations [23]. Therefore, dose adjustment and deprescribing of inappropriate medications are important components of medication reviews for older patients [24]. Medication optimization in older patients is challenging due to the presence of comorbidities and individual variations in age-related physiological changes including pharmacokinetic, pharmacogenetic, and pharmacodynamic responses to medications [25, 26].

Pharmacist-led medication reviews have been proposed as an important component of optimizing medication prescribing [27], but there is conflicting evidence regarding the impact and efficacy of medication reviews for older patients in the ED [28–32]. International studies have shown that collaboration between pharmacists and physicians in multidisciplinary hospital teams can decrease inappropriate prescribing among older patients in a transitional setting [33–35]. We have previously shown that a medication review in the ED is feasible and led to increased medication appropriateness between admission and discharge in 64% of the patients [36]. However, this finding has never been demonstrated among older patients in the ED using a randomized controlled trial. Therefore, we aim to use a randomized controlled trial to determine the efficacy of a medication review (MED intervention) in a cohort of acutely admitted older patients both without and with risk of malnutrition.

#### Accuracy of estimated glomerular filtration rate in older medical patient

Another challenge during medication optimization is the accurate assessment of renal function [37, 38]. This is important because approximately 40% of all medications require dose adjustment according to renal function [39]. In clinical practice, renal function is typically determined with estimated glomerular filtration rate (eGFR) based on the measurement of serum creatinine. However, serum creatinine is heavily dependent on non-GFR determinants such as muscle mass, nutritional status, and sex, which can result in inaccurate eGFR and, therefore, inappropriate prescribing of renally cleared medications among older patients [40, 41]. Alternative markers of kidney function such as cystatin C, beta-trace protein (BTP), and beta-2-microglobulin (B2M) are less dependent on muscle mass, age, and sex compared to serum creatinine and may be more appropriate in older patients [40–42]. However, these novel biomarkers may be affected by other non-GFR determinants, such as inflammation, and there is a lack of knowledge regarding their impact among acutely admitted older patients [40, 41]. Therefore, we aim to determine which kidney marker or combination of markers is most accurate for older medical patients, as well as the impact of non-GFR determinants on this accuracy.

#### Malnutrition and nutritional intervention

Malnutrition can lead to decreased physical function and quality of life, rehospitalization, and death [43–45]. Malnutrition among older adults often has a multifactorial etiology [46] including dysphagia [47, 48], poor appetite [49–51], dry mouth [52], problems in the oral cavity [48, 50], polypharmacy [49, 53], hospitalization [50], decreased cognitive capacity, and decreased ability to perform activities of daily living such as cooking, grocery shopping, and eating [48]. Medications can also contribute to the development of malnutrition, as they can induce dry mouth [54], nausea [55], constipation [56], diarrhea [56], and anorexia [57].

The European Society for Clinical Nutrition and Metabolism (ESPEN) recommends that nutritional interventions in older persons should be individualized, multimodal, multidisciplinary, and comprehensive to improve the quality of life. They also recommend that potential causes of malnutrition are systematically identified and eliminated if possible [58]. To our knowledge, however, there is no evidence regarding the effect of a multimodal and transitional nutritional intervention in acutely admitted older medical patients. Therefore, we aim to test the effect of an individualized multimodal and transitional nutritional intervention (MULTI-NUT-MED intervention) among acutely admitted older medical patients using a randomized controlled trial.

### Aims

The study involving acutely admitted older medical patients has three primary aims (1) to determine whether a medication review intervention is superior to standard care in improving medication appropriateness from admission to 8 weeks after discharge, (2) to determine the accuracy of eGFR equations based on various biomarkers compared to mGFR at a single-time point, and (3) to determine whether an individualized multimodal and transitional nutritional intervention is superior to standard care in improving the quality of life from admission and 16 weeks after discharge.

The study also has the following secondary aims which are related to the effect of the intervention: (1) to measure differences in polypharmacy, PIM use, and assessment of underutilization between the MED-intervention and standard care from admission to 8 weeks after discharge and (2) to measure of the differences in body weight, protein- and energy intake, mobility, cognition, frailty, the intestinal microbiome, and health care costs between the MULTI-NUT-MED intervention and standard care from admission to 8 and 16 weeks after discharge and 1 year after discharge (only health care costs).

Secondary aims, which are based on baseline data or data from the control group, include assessment of (1) how information from a broad pharmacogenetic test can potentially improve medication prescribing appropriateness; (2) how polypharmacy, PIMs, nutritional status, frailty, and inflammation modify the effect of the MED-intervention on prescribing appropriateness; (3) how non-GFR determinants affects eGFR and how the choice of eGFR equation affects medication prescribing; (4) how the choice of malnutrition classification criteria affects the prevalence of malnutrition; and (5) how different definitions of frailty affect the distribution of patients classified as frail or sub-frail as well as the responsiveness of these methods.

## Methods: participants, interventions, and outcomes

### Overview and trial design

**Fig. 1:**
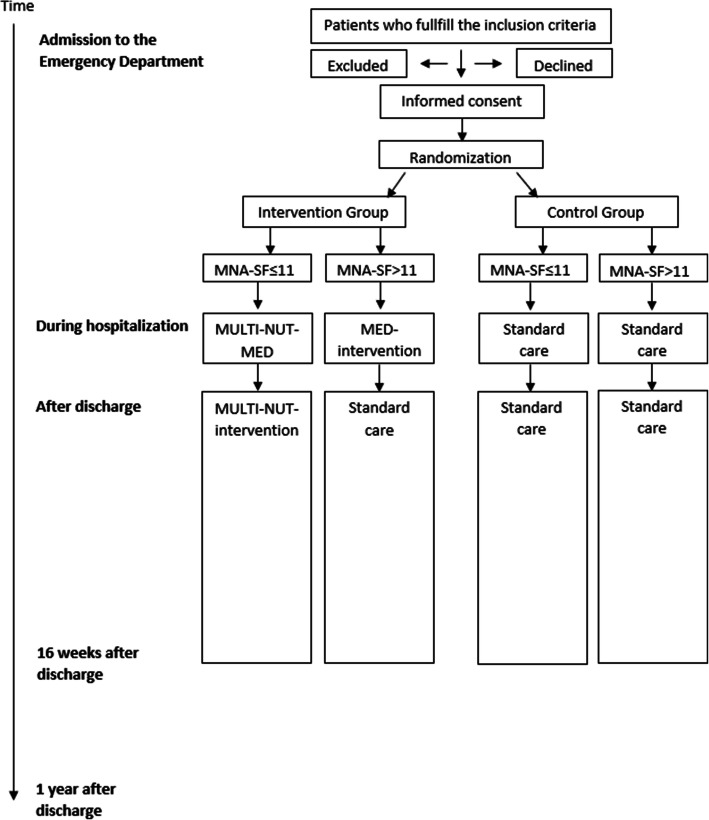
Overview of the trial design. Mini Nutritional Assessment-Short Form (MNA-SF), Multidisciplinary (MULTI), Nutritional (NUT), and Medication (MED)

**Fig. 2:**
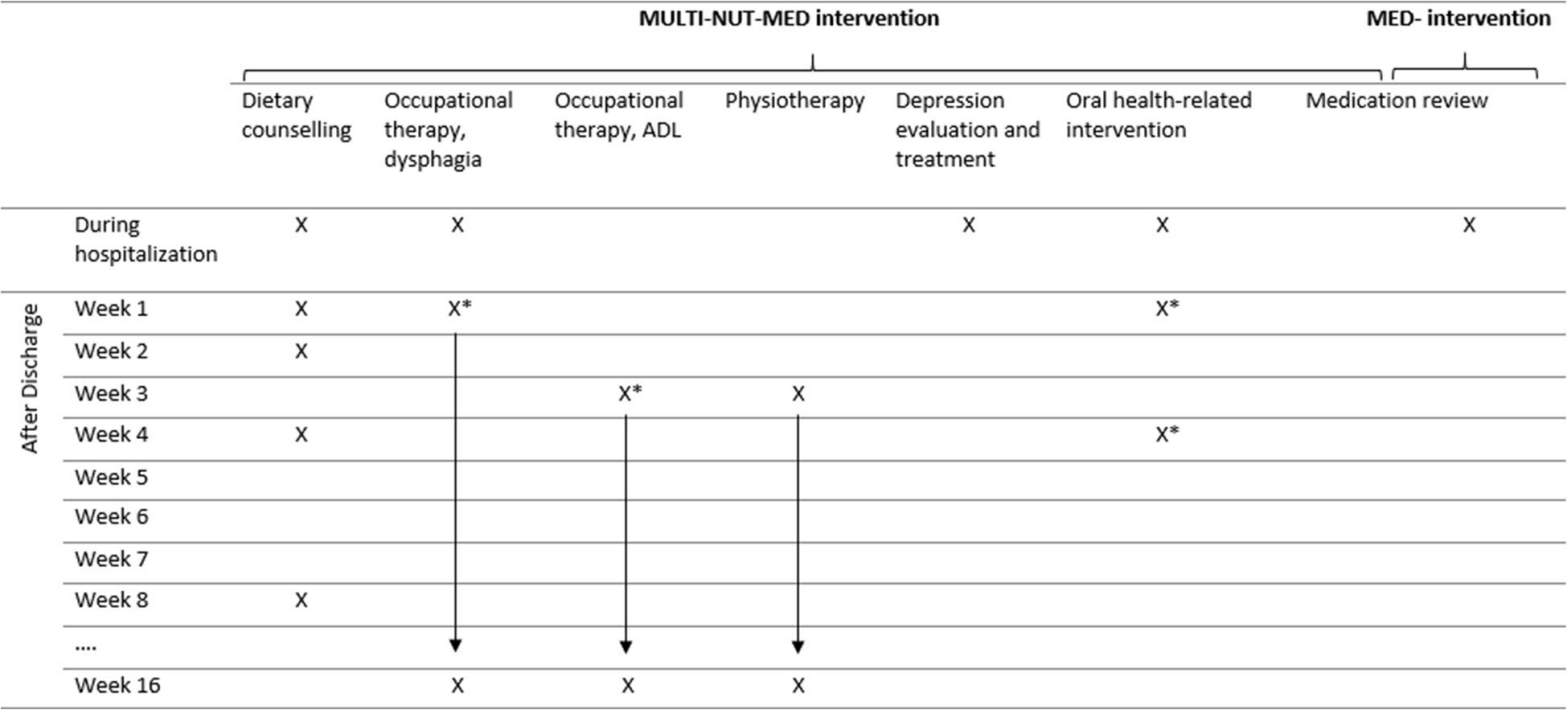
Schematic overview of the MULTI-NUT-MED and MED intervention elements and their timeframe. X indicates the timepoint of the intervention element. *These interventions are provided on an individually assessed need. Thus, only the possible timespan of the intervention is indicated. Abbreviations: Multidisciplinary (MULTI), Nutrition (NUT), Medication (MED), and Activities of Daily Living (ADL)

The OptiNAM trial is a single-center block randomized, controlled, observer-blinded, superiority, and explorative trial with two parallel groups. The trial starts upon admission to the ED and includes follow-up visits at 8 and 16 weeks after discharge and a telephone interview at 1 year after discharge in participants with MNA-SF≤11 to collect EuroQol-5D-5L. Figures 1 and 2 and Table 1 provide schematic overviews of the trial design. The trial was initiated on October 15, 2018, and adheres to the Standard Protocol Items: Recommendations for Interventional Trials (SPIRIT) Statement [59]. Trial registration data (Table 1s) and a SPIRIT checklist are provided in the Supplementary material.

**Table 1:** A schematic overview of the study design. Definition of timepoints: t_1_: Within 36 h after admission the ED, t_a_: Immediately after data collection upon enrollment has ended, t_GFR_: During hospitalization or up to 2 weeks after discharge, t_2_: 8 weeks after discharge, t_3_: 16 weeks after discharge, t_1y_: 1 year after discharge. Abbreviations: *GFR* glomerular filtration rate, *MULTI* multidisciplinary, *NUT* nutritional, *MED* medication

Timepoint	Enrollment	Allocation	Post allocation			Close-out
	t_1_	t_a_	t_GFR_	t_2_	t_3_	T_1y_
**Enrolment**						
Eligibility screening	x					
Informed consent	x					
Allocation		x				
Nutritional screening	x					
**Interventions**						
MULTI-NUT-MED intervention		x			x	
MED intervention		x				
**Data collection**						
*Descriptive variables*	x					
**Primary outcomes**						
*Health related quality of life*	x			x	x	x
*Medication appropriateness*	x			x	x	x
*Kidney Function*			x			
**Confirmative outcomes**						
*Underutilization of medication*	x			x	x	
*Energy and protein intake*	During admission			x	x	
*Mobility*	x			x	x	
*Activities of daily living*	x			x	x	
*Well-being*	x			x	x	
*Frailty*	x			x	x	
*Anthropometry*	x			x	x	
*Blood pressure and heart rate*	x			x	x	
*Mortality*					x	x
*Cognition*	x			x	x	
*Depression*	x			x	x	
*Blood samples*	x		x	x	x	
*Health economy*	x				x	x
**Explorative outcomes**						
*Gene variations*	x					
*Body composition*	x		x	x	x	
*Nutritional status*	x			x	x	
*Intestinal microbiota*	x			x	x	

### Setting

The tax-funded Danish health care system is based on the principles of free and equal access to health care for all citizens. In Denmark, there are approximately 1 million acute hospital admissions each year, of which 46% are older persons [1]. This trial is conducted at Copenhagen University Hospital, Hvidovre, Denmark, which covers 10 municipalities with approximately 550,000 citizens and has about 14,000 medical admissions each year of which 85% are acute admissions [36]. The ED has a 29-bed-medical-ward handling acute medical admissions, and a separate emergency room (ER) handling minor injuries and traumas. Patients are referred to the ED by a general practitioner (GP), medical helpline, or emergency phone call. From the ED, a patient can either be discharged or transferred to a specialized medical ward. The hospital does not have a geriatric ward, but the ED is permanently staffed by geriatricians and clinical pharmacists. Dieticians, occupational therapists and physiotherapists are available for consult. Dentists are not part of the normal hospital staff.

### Eligibility criteria, recruitment, and consent

Patients are eligible for inclusion if they are ≥65 years of age, acutely admitted to the ED, community-dwelling, and residing in one of the following municipalities: Hvidovre, Copenhagen (Districts of West and South Western Copenhagen), or Brøndby. Exclusion criteria are inability to understand Danish, inability to cooperate physically (e.g., hearing or speech impairment), or cognitively (e.g., dementia or unconsciousness), isolation room stay, not Caucasian, and admission due to suicide attempt or terminal illness.

Participants are recruited up to 36 h after admission. Each morning (Monday through Thursday), patients are assessed for eligibility from a randomized computer-generated list of all patients in the ED. Eligible patients are informed about the trial both verbally and in writing by a team of seven researchers and are given the possibility of a reflection period of 24 h to consent to participation. Participants are given the option to consent separately to GFR measurement and pharmacogenetic test. After obtaining written informed consent, each participant is randomized to either the intervention group or standard care, and baseline data are collected. After baseline data are collected, each participant is informed about the result of the randomization by staff performing the intervention.

The following strategies are implemented to minimize the effect of staff shortages on patients recruitment: (1) on days with insufficient hospital staff available to perform the MULTI-NUT-MED intervention, only patients with a Mini Nutritional Assessment Short-Form (MNA-SF) [60] score >11 are recruited (to the MED intervention only); (2) on days with insufficient community-based staff available to perform future nutritional intervention (e.g., due to vacation or long term illness), patients from these municipalities are not assessed for eligibility; (3) if more than 35% of the first 50 included patients (*n* = 50) have an MNA-SF score >11, then the project leaders can decide to exclusively recruit patients with an MNA-SF score ≤11 2 days of each week in order to achieve a sufficient number of participants with an MNA-score ≤11 (see the “Sample size” section). The expected recruitment rate is two participants per week for 100 weeks.

### Standard care

The intervention is given in addition to standard care. The control group is offered standard care only. Standard care was chosen as the comparator as this is what needs to be improved.

Medication reconciliation is performed by a clinical pharmacist within 24 h of ED admission for all patients [61]. If available, an initial medication list is obtained from the admitting ED physician. This initial list is compared with the hospital’s electronic patient record system and Shared Medication Card, a central containing information about all medications prescribed and dispensed within the previous 2 years [62]. Each participant is then asked to confirm the use of each medication. If a participant is unable to provide reliable information, then the medication list is confirmed with a caregiver (e.g., family member, GP, home nurse, nursing home staff, or community pharmacy). The final medication list is documented in the electronic patient record along with any discrepancies between the initial and final medication list. The final medication list is communicated to the providing geriatrician, who updates the participant’s electronic prescriptions accordingly.

According to the regional nutritional guideline in the Capital Region of Denmark, all patients with an expected hospital stay of more than 24 h should be screened for nutritional risk during the first 24 h of hospitalization and have treatment initiated when relevant [63]. All trial participants are offered the same foods and nutritional products during hospitalization, except for the protein supplement P-Boost®, which is only offered to participants in the intervention group (see the “Dietary Counseling” section).

All municipalities offer food service (paid by the recipient) and home care meal support. None of the municipalities have systematic identification of malnutrition. The municipality of Hvidovre offers home visits from a dietician for citizens who experience unintentional weight loss or declining functional ability or if the municipality is informed of a citizen at risk of malnutrition during hospitalization. The dietician offers unlimited dietary counseling until the goal of counseling is reached, or the counseling is deemed to be unfruitful. The municipality of Copenhagen offers nutritional advice from a nurse to citizens identified as having malnutrition by care staff. The municipality of Brøndby offers dietary counseling from a community-based dietician to citizens with malnutrition who are identified by care staff. Due to the variation between the municipalities, randomization is stratified by the municipality.

### Interventions

The intervention is individualized: participants in the intervention group without malnutrition (MNA-SF >11) only receive the MED intervention, and participants in the intervention group with or at risk of malnutrition (MNA-SF ≤11) receive the MULTI-NUT-MED intervention (see Fig. 1 and Table 1).

#### Medication review intervention (MED intervention)

After the final medication list is obtained and baseline data are available, each participant is given a structured, patient-oriented medication review by the clinical pharmacist. All medications are reviewed to evaluate the (1) agreement with current national guidelines regarding choice of medication, dose, and dosing interval; (2) whether the goal of treatment has been met; (3) presence of nausea, constipation, diarrhea, loss of appetite, or dry mouth related to use of the medication; and (4) whether there is inadequate treatment of any current diagnoses or conditions. PIMs are identified using the Screening Tool of Older Persons’ Prescriptions criteria version 2 [64]. Prescribing recommendations based on eGFR are obtained from Renbase® [65] and “pro.medicin.dk,” a database containing information on national medication and treatment guidelines [66]. Medications are considered “renal risk medications” if dose adjustments are recommended at eGFR ≤90 mL/min/1.73 m^2^. Drug interactions are determined using the national database “interaktionsdatabasen.dk” [67]. Finally, each prescribed medication is assessed for appropriateness based on the indication for treatment, recommended dose, adverse drug reactions, therapeutic duplication, dosing interval, formulation and strength, drug interactions, contraindications, precautions, and specific participant characteristics. The clinical pharmacist documents any recommended changes to the participant’s medication list in the electronic patient record. These recommendations are then reviewed by a geriatrician, who decides whether to accept, reject, or alter each recommendation in collaboration with the clinical pharmacist. Any medication changes made by the geriatrician are documented, in the electronic patient record and implemented in the ED, accepted but not implemented in the ED, communicated to a specialized hospital ward and/or the participant’s GP, or rejected [36].

#### Multidisciplinary nutritional intervention (MULTI-NUT-MED intervention)

**Fig. 3:**
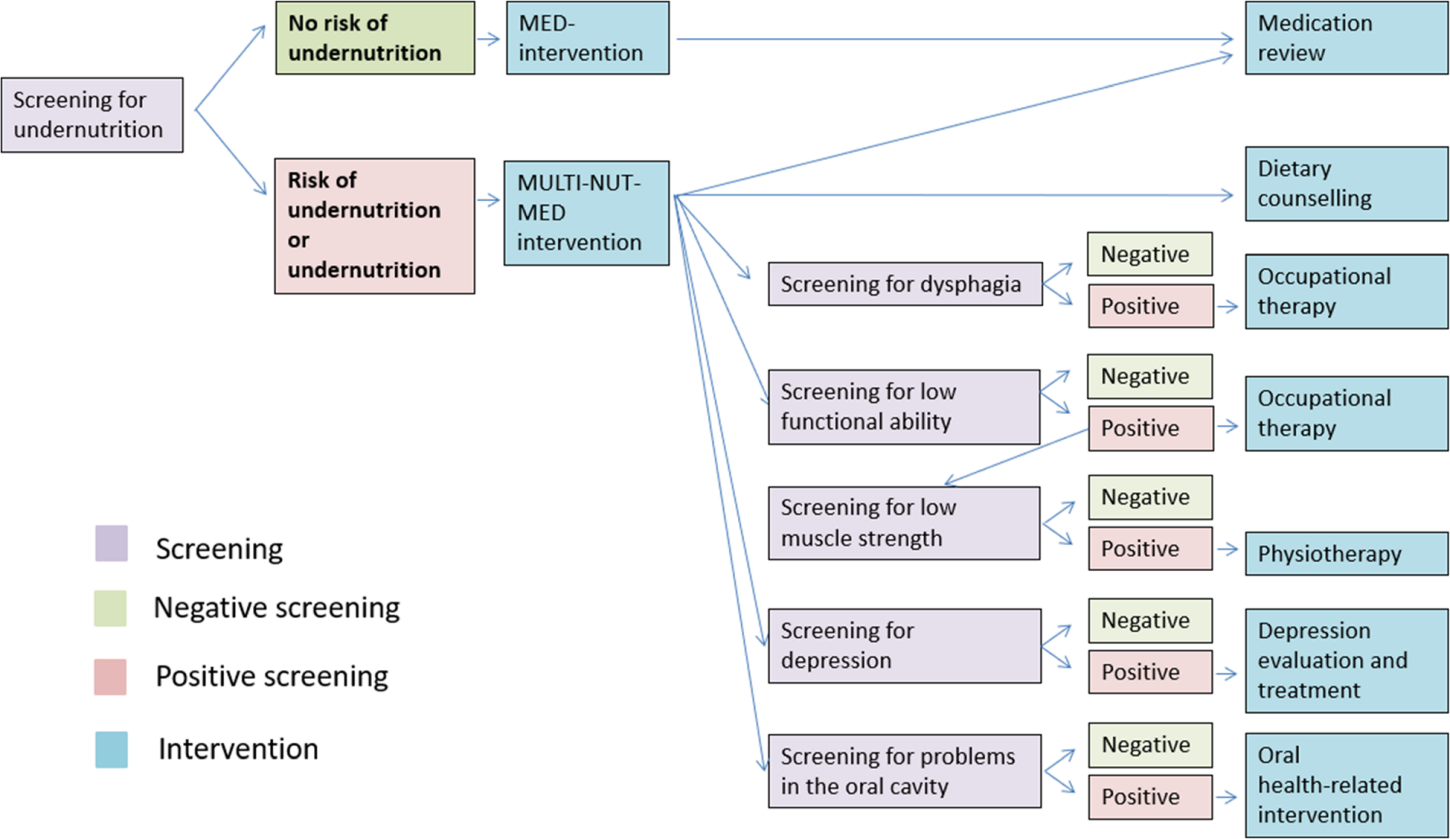
Overview of interventions offered to participants in the intervention group. Details of the screening procedures and interventional content can be found in the section “Medication review intervention” and “Multidisciplinary Nutritional intervention”. Abbreviations; Multidisciplinary (MULTI), Nutritional (NUT), and Medication (MED)

In addition to the MED intervention described above, the multidisciplinary nutritional intervention consists of an individualized multimodal and transitional intervention that includes dietary counseling, occupational therapy, physiotherapy, evaluation and treatment of depression, and an oral health-related intervention. The choice of intervention is based on screening tests performed during the baseline data collection (see Figs. 2 and 3).

#### Dietary counseling

Dietary counseling is provided both during hospitalization, by research dieticians, and after hospital discharge by community-based dieticians or by hospital-based research dieticians if community-based dieticians are not available. The goal of dietary counseling is to ensure that each participant receives a diet that covers the energy and protein requirements to either maintain their body weight (if BMI≥18.5) or gain body weight (if BMI <18.5). Both the content of the dietary counseling and the structure of the home visits after discharge are inspired by Lindegaard et al. [68, 69].

##### During hospitalization

Immediately after randomization, the research dietitian creates a dietary plan in collaboration with the participant for the participant to follow during hospitalization. This dietary plan is based on foods and nutritional products available at the hospital as well as the participant’s nutritional needs [70], self-reported food preferences, and ability to chew (oral or dental pain and masticatory difficulties) and swallow (evaluated by the EAT-10 score [71, 72] and an occupational therapist).

Foods and nutritional products available at the hospital include a fixed menu in the ED or an *a la carte* menu in specialized wards, energy- and protein-enriched or texture-modified menus, oral nutritional supplements (ONS), and a selection of beverages and in-between meals. Participants in the MULTI-NUT-MED intervention may also receive P-Boost®(Adosan), a fluid protein-only supplement.

Daily dietary intake is recorded in a food diary by the participant and validated by the research dietician the following day. If a food diary is incomplete, then the research dietician performs a 24-h dietary recall [73]. Both dietary intake and dietary plans are entered in VITAKOST [74]. If 100% of energy and protein and energy requirements is not achieved on a given day, then the dietary plan is adjusted for the following day. If >75% of protein and energy requirements is not achieved for 2 days in a row, then tube feeding or parenteral nutrition is advised in collaboration with the medical doctor.

##### After discharge

Prior to discharge, a dietary plan for the first week after discharge is created based on the participant’s food preferences, nutritional needs (evaluated by the Nordic Nutrition Recommendations [75] with an optional stress factor), ability to chew (pain in their mouth, difficulties chewing) and swallow (evaluated by the EAT-10 score [71, 72] and an occupational therapist), and limitations in grocery shopping, cooking or eating (evaluated by the Functional Recovery Score (FRS) [76]). If ONS is a part of the dietary plan after discharge, then the participant is provided with an ONS prescription, which can be filled at the pharmacy with a 60% price reduction. At discharge, the dietary plan is provided to both the participant and community-based dietician.

Participants are offered 1-h home visits by the community-based dietician at 1, 2, 4, and 8 weeks after discharge to evaluate changes in body weight and energy and protein intake and to adjust the dietary plan if needed. If a participant is readmitted within 16 weeks of discharge, they are offered dietary counseling during rehospitalization.

##### Occupational therapy—dysphagia

Participants with an EAT-10 score [71, 72] ≥3 at baseline are offered an evaluation by a hospital-based occupational therapist to identify the presence of dysphagia. If the participant is discharged prior to the hospital-based evaluation, then a community-based occupational therapist performs the evaluation after discharge.

If the initial evaluation of dysphagia reveals areas for potential treatment, then an intervention to improve and secure swallowing safety and efficiency is initiated. Both the evaluation and treatment are performed according to the principle of Facial Oral Tract Therapy (F.O.T.T.®) and follow prespecified algorithms [77]. Any required modifications to food and beverage consistency are coordinated with the research dietician during hospitalization and communicated to community-based staff performing the intervention after discharge. If any additional interventions for dysphagia are still required at the time of discharge, then the participant is offered home visits by a community-based occupational therapist as needed for 2 h per week for 16 weeks after discharge. All evaluation results and treatment plans are communicated to the community-based occupational therapist upon discharge. The intervention is terminated when an individualized goal of intervention is reached or after 16 weeks.

##### Occupational therapy—activities of daily living

Participants with a FRS [76] ≤2, at baseline, in one or more of the following categories: grocery shopping, cooking, or eating (not including dysphagia) are offered an intervention by a community-based occupational therapist starting within two weeks after discharge.

The choice of intervention is based on the Assessment of Motor and Process Skills (AMPS) [78], which evaluates the quality of performed activities of daily living (ADL) according to the degree of physical effort, efficiency, safety, and independence. Following this assessment, an appropriate activity-based intervention is planned by the occupational therapist in collaboration with the participant according to the principles of the Occupational Therapy Intervention Process Model (OTIPM) [79]. If there is potential to maintain or regain functional ability, then the participant is offered 1-h home visits by a community-based occupational therapist during the first 16 weeks after discharge for up to 7 home visits or until the goal of intervention is reached. In collaboration with the participant, the therapist sets the goal of maintaining or regaining functional ability in groceries shopping, cooking, and/or eating. If there is no potential to maintain or regain functional ability, then the intervention is terminated after the first home visit.

##### Physiotherapy

Participants who screen positive for low functional ability (i.e., FRS scores ≤2 in grocery shopping, cooking, or eating) are also evaluated for lower extremity strength during baseline data collection. Decreased muscle strength is defines defined as <5 repetitions on 30 s sit-to stand test [80, 81] or 5–8 repetitions on 30 s sit-to stand test in combination with a 4-m gait speed [82] below 0.6 m/s [81]. Participants with decreased muscle strength at baseline are re-evaluated at 2–3 weeks after discharge by a community-based physiotherapist. Participants who still have decreased muscle strength at this timepoint are offered two weekly training sessions [83] by a community-based physiotherapist for 16 weeks after discharge. The training sessions are approximately 1-h in duration and take place either in the participants home or at a training facility. The training is intended to improve the participant’s ability to maintain independent ADL by combining exercises for strength, endurance, and balance [84]. The specific exercises include a progressive sit-to-stand exercise as reported by Pedersen et al. [85], a static balance exercise, where the participant pokes for the hands of the physiotherapist to train the participant’s agility [86] by altering the base of support for 3 sets of 10–60 s, an up-and-down-from-floor exercise, a stair climbing exercise till fatigue or for a maximum of 10 min and a walking exercise, aiming to increase step counts by 10% each week [87], based on the walking capabilities of the participant. Participants with unsafe walking abilities will have supervised walking for 10 min with the physiotherapist. All exercises follow prespecified algorithms for training progression and are adjusted accordingly for each session. During the intervention, participants are also weighed once each week by the physiotherapist. If a participant has > 1 kg weight loss since the first training session, then training is paused, and a dietician provides dietary counseling until body weight is stabilized, at which point training is resumed.

##### Evaluation of depression and treatment

Participants are screened during baseline data collection for depression using the Mini Geriatric Depression score [88]. Participants with a Mini Geriatric depression score ≥2 are subsequently screened with the Geriatric Depression Score-15 (GDS-15) [89]. Participants with a GDS-15 ≥5 are offered evaluation and treatment for depression by a geriatrician during hospitalization. After discharge, the geriatrician can follow up on treatment by phone. If the participant is discharged before evaluation and treatment by the geriatrician, then they are recommended to see their GP.

##### Oral health-related intervention

Participants are screened during baseline data collection for oropharyngeal dysfunction. Participants who report oral pain, masticatory difficulties, and/or xerostomia (Summated Geriatric Xerostomia Inventory≥8) [90] are offered a clinical oral evaluation including diagnosis of potential oral and/or dental disease conditions by a research dentist. This evaluation includes an interview on oral symptoms and an evaluation of the dental, periodontal and oral mucosal status, the oral hygiene status, and functional occlusion as well as the function of dentures (partial or full, if present). In case of suspicion of oral candidiasis, an oral smear is performed, and antifungal treatment initiated if necessary. Overall, if dental treatment is required, the participant is advised to attend to their dentist. If the oral hygiene is insufficient and the participant displays signs of gingivitis and periodontitis and/or impaired salivary gland function, they are offered two home visits from a dental hygienist after discharge. This intervention includes instruction and motivation in order to improve the oral hygiene and thereby oral health. Participants with insufficient oral hygiene are also provided with an electrical toothbrush.

#### Training of staff performing the interventions

All clinical pharmacists are educated and certified by The Capital Region Pharmacy, Herlev, Denmark, to perform medication reconciliation and medication reviews. All clinical pharmacists are provided 1 h of instruction about the intervention by CT, AKB, or the primary investigator MBH. Additional instruction may be provided by MBH as needed during the trial.

Both hospital- and community-based dieticians are provided one hour of instruction about the intervention by the primary investigators, ALA, or RLN. This instruction reviews standardized procedures for data collection and dietary interventions and may be repeated if deemed necessary. Hospital-based dieticians are also provided supervised training in dietary interventions by ALA, RLN, or already trained dieticians until deemed ready to perform the intervention unsupervised. Additional instruction may be provided by ALA or RLN as needed during the trial.

Both hospital- and community-based occupational therapists and physiotherapists are provided 1 h of instruction about the intervention by primary investigators ALA or RLN. This instruction reviews standardized procedures for data collection and therapy interventions and may be repeated on an individual basis upon request.

#### Compliance

Participant compliance is defined separately for each element of the intervention. Compliance to medication review is defined as having received a medication review that is evaluated and effectuated by a geriatrician. Compliance with dietary counseling is defined as having counseling during hospitalization and ≥3 home visits after discharge. Compliance with physiotherapy is defined as having received ≥75% of training sessions. Compliance with occupational therapy is defined as having received ≥75% of the home visits for dysphagia and ≥75% of home visits for ADL if deemed relevant by the occupational therapist. Compliance to evaluation and treatment of depression is defined as having received an evaluation by the geriatrician. Compliance with oral health-related intervention is defined as having received an evaluation by a dentist and ≥1 home visit by a dental hygienist if deemed relevant by the dentist.

#### Adherence

Recruitment, data collection, and interventions are all performed either in the hospital (during hospitalization) or in the participant’s home (after discharge). This strategy is performed to maximize patient recruitment, data collection, and compliance to the intervention while minimizing participant transportation costs and efforts. Adherence to the intervention is registered and evaluated but not monitored during the trial.

### Outcomes

Trial outcomes as well as timepoints and collection methods for each outcome are shown in Table 2 and Fig. 3.

**Table 2:** An overview of all outcomes in the OptiNAM-trial

Variable	Instrument	Time point
Primary outcomes		
Health-related quality of life	EuroQol-5D-5L is a self-reported questionnaire comprised of 5 questions concerning: mobility, self-care, usual activities, pain/discomfort and anxiety/depression, and a visual analog scale (VAS-scale). Each question has 5 response categories, ranging from having no problems to being unable. The responses are converted into an index value, reflecting the health status compared to the reference of the general population (norm data) [91, 92]. The VAS-scale ranges health at a scale from 0 (worst health you can imagine) to 100 (best health you can imagine).	t_1_, t_2_, t_3_, t_1y_
Medication appropriateness	Medication Appropriateness Index-score (MAI-score) consists of 10 criteria addressing different aspects of each prescription, including indication, effectiveness, dose, direction, practical direction, drug–drug interaction, drug–disease interaction, duplication, duration of therapy, and cost [93]. Each criterion has operational definitions that instruct the evaluator to rate a medication as (A) appropriate, (B) marginally appropriate, (C) inappropriate, or (Z) do not know [94]. Each of the criteria is assigned a score between 0 and 3 according to a standardized protocol. Each prescription can obtain a score between 0 and 18 [95], where 0 represents appropriate prescribing and higher scores indicate a greater degree of inappropriateness.	t_1_, t_2_, t_3_
Kidney function	The glomerular filtration rate (GFR) is determined according to the single-injection plasma clearance method [96, 97]. Radioactivity is measured in multiple venous blood samples obtained between 180 and 300 min after a single intravenous injection of 40 MegaBecquerel ^99m^Technetium–diethylenetriaminepentaacetic acid (^99m^Tc-DTPA) and GFR is calculated from the plasma disappearance curve of ^99m^Tc-DTPA.	t_GFR_
Confirmative outcomes		
Underutilization of medication	The Assessment Of Underutilization (AOU) index identifies omitted medication prescribing despite being indicated [98]. The evaluation requires a full medication list as well as a list of established medical conditions to apply one of three ratings for each condition: (A) no omission, (B) marginal omission, or (C) omission of an indicated medication without contraindication [94].	t_1_, t_2_, t_3_
Pharmacogenetic	DNA material is collected by a buccal swab. The genetic test involves variations in 14 genes and copy variants responsible for drug transport and metabolism of more than 140 commonly prescribed medications. The included genes are Cytochrome P450 (CYP) 1A2, 2B6, 2C19, 2C8, 2C9, 2D6, 3A4, 3A5, Dihydro-pyrimidine Dehydrogenase, Opioid Receptor Mu 1, Solute Carrier Organic Anion Transporter Family Member 1B1 (SLCO1B1), UDP glucuronosyltransferase family 1 member A1, UDP-glucuronosyltransferase 2B15, and Vitamin K epOxide Reductase Complex subunit 1 [99]. After genomic data translation, a personalized evidence-based report is generated based on recommendations from the Food and Drug Administration drug labels, PharmGKB and Clinical Pharmacogenetics Implementation Consortium guidelines [100, 101]. The Pillcheck software (GeneYouln Inc., Toronto/Ontario, Canada) is used for the categorization of metabolic status classes linked to evidence-based recommendations for drug prescribing, and for identifying medications that may cause reduced clinical efficacy or significant drug reactions at standard starting doses [26].	t_1_
Dietary intake	Macronutrient intake is assessed based on validated dietary records or 24-h recalls and calculated with the software VITAKOST (VITAKOST ApS, Kolding, Denmark) [74]. During hospitalization, daily dietary records are kept by the participant and validated by a dietician (only two days for participants in the control group). At follow-up visits in weeks 8 and 16, a 3-day dietary record is validated by a dietician using household measures and photo material [102] to estimate portion sizes. In case of missing dietary records, a 24-recall [73] is performed instead, which also is validated by a dietician using household measures and photo material [102].	During hospitalization , t_2_, t_3_
Mobility	Maximal hand grip strength is measured with a hand dynamometer (Saehan, Digi-II) in three attempts [103]. If the last attempt is the peak value, another two attempts are given as described by Bodilsen et al. [104]. A 30-s chair-stand-test measures the number of full rises from a sitting position in a chair without support from the arms performed in 30 s [80]. A 4-m walking test measures habitual walking pace (m/s) on a 4-m long track [105]. The De Mortons Mobility Index (DEMMI) measures the ability to perform mobility tasks of increasing difficulty, from transferring in bed to jumping. DEMMI provides a crude score from 0 to 19, which is converted to a DEMMI-scale score from 0 to 100, where 100 is the highest level of mobility [106–109]. ActivPAL® is an accelerometer (PAL Technologies Ltd., Glasgow, UK) [110], mounted mid-thigh which measures time spend lying/sitting, standing, and walking and daily number of steps.	t_1_, t_2_, t_3_ 1^th^ week after discharge, t_2_, t_3_
Activity of daily living	The Functional Recovery Score measures the degree of dependency in 11 different ADL [76].	t_1_, t_2_, t_3_
Well-being	The 5-item World Health Organization Well-Being Index measures well-being on a scale from 0 to 100, where 100 is the highest level of well-being [111].	t_1_, t_2_, t_3_
Frailty	Fried’s frailty phenotype evaluates frailty on 5 aspects: measured Hand Grip Strenght and walking pace, self-reported physical activity level, exhaustion, and weight loss [112]. The FRAIL questionnaire [113] includes questions concerning fatigue, capability of stair climbing, walking distance, multi morbidity, and weight loss. The Frailty Index, FI-Outref [114], is calculated by using the number of admission laboratory test results being outside the reference interval.	t_1_, t_2_, t_3_
Anthropometry	Body weight is measured with or without shoes and in light clothing. Waist circumference is measured in a standing position after a normal exhalation. Self-reported height is registered.	t_1_, t_2_, t_3_
Body composition	Total and segmented lean body mass and bone mineral content are measured with whole-body Dual-energy X-ray Absorptiometry (DXA) (GE Lunar Prodigy Primo, GE Healthcare Technologies, Madison, Wisconsin, US). DXA-scan is a clinical standard and validated to assess body composition [115]. Total and segmented body fat, fat-free mass, soft lean mass, bone mineral content, and intra- and extracellular water are measured using Bioelectrical Impedance Analysis (BIA) performed with InBody S10 [116]. Standardization, regarding fasting, physical activity, and urination prior to the measurement are not possible due to the acute setting. Information on these parameters is therefore collected prior to the measurement.	t_GFR_ During admission, t_1_, t_2_, t_3_ and t_GFR_
Blood pressure and heart rate	In a sitting, relaxed position blood pressure and heart rate are measured with a Microlife, BP A3L Comfort, automatic monitor, 3 times in a row on the right upper arm, with a break of 30 s in between measurements.	t_1_, t_2_, t_3_
Mortality	The Danish Register of causes of death [117] is a national registry where the cause of death noted in the medical evaluation after death is gathered.	t_1_, t_2_, t_3,_ t_1y_
Cognition	The trail making test [118] assesses the time it takes the participant to draw a line between 25 consecutive numbers that are scattered randomly on a piece of paper and are recorded. In the Digit Symbol Modalities test [119], the participant fills in as many as possible digits corresponding to a symbol in 90 s. The number of correctly filled in digits is recorded. In Hopkins Verbal Learning Test Revised [120], 12 words are read out loud, and the number of recalled words are recorded. Further, a list containing the 12 words and other words is read out loud, and the correctly and incorrectly recognized words are recorded. A delayed recall is of the 12 words is furthermore performed. The Mini-Mental State Examination [121] consists of 11 questions and tasks and provides a score between 0 and 30. The Orientation-Memory-Concentration test [122] consists of three questions concerning time and place, two tasks of concentration, and one task on memory.	t_2_, t_3_ t_2_, t_3_ t_2_, t_3_ t_2_, t_3_ t_1_, t_2_, t_3_
Depression	The Mini-Geriatric Depression Score [88] measures the need for medical evaluation of depression with 5 yes/no questions.	t_1_, t_2_, t_3_
Nutritional risk status	The Mini Nutritional Assessment- short form [60] is a screening tool, consisting of 6 questions concerning food intake, weight development, mobility, acute illness, cognition, and BMI. The result classifies the patient as malnourished, at risk of malnutrition or having normal nutritional status. The Nutritional Risk Screening-2002 [123] classifies the patient’s risk of malnutrition based upon a primary and a secondary screening. The primary screening consists of 4 yes/no questions. If one answer is yes, the secondary screening is performed. The secondary screening evaluates the degree of illness and malnutrition. The Eating Validation Scheme [124] is a screening tool that classifies a person as having no risk of malnutrition, at risk of malnutrition or in need of nutritional intervention based on evaluation of eating habits, weight development, and risk factors of malnutrition. The Eating Symptom Questionnaire [125] clarifies if 13 different difficulties (e.g. nausea, dry mouth, pain) are related to the development of malnutrition. The Simplified Nutritional Appetite Questionnaire [126] identifies persons with anorexia and risk of weight loss by asking 4 questions on appetite, fullness, taste, and number of daily meals. The Global Leadership Initiative on Malnutrition (GLIM) criteria [127] is a consensus-based 2-step approach for malnutrition diagnosis. The first step is screening for malnutrition and the second step is the assessment of malnutrition diagnosis and grading of malnutrition severity. Malnutrition is diagnosed if at least one phenotypic criterion (non-volitional weight loss, low body mass index, and reduced muscle mass) and one etiologic criterion (reduced food intake or assimilation and inflammation or disease burden) are present. The European Society of Clinical Nutrition and Metabolism statement [128] is a European consensus-based set of criteria for the diagnosis of malnutrition. After a positive screening result for malnutrition, malnutrition is diagnosed based on low body mass index or on unintentional weight loss together with low BMI or low fat-free mass index	t_1_, t_2_, t_3_
Dysphagia	The Eating Assessment Tool-10 (EAT-10) [71, 72] is a 10-question questionnaire which identifies persons with a need for evaluation of dysphagia.	t_1_, t_2_, t_3_
Intestinal microbiome	All participants at risk of malnutrition or with malnutrition according to the nutritional screening tool MNA-SF will collect a fecal sample using EasySampler [129]. Samples collected during hospitalization are frozen as fast as possible at −80°. If collected in the home of the participant, the sample is frozen as fast as possible in the participants own −18° freezer for maximally 3 days. The samples are transferred in a cooler bag with freezer elements to the −80° freezer (Biobank). At the time of collection, the participant reports where the sample belongs on the Bristol Stool Scale [130] and their use of antibiotics.	t_1_, t_2_, t_3_
Blood samples	Blood samples are analyzed for: Alanine aminotransferase, albumin, basic phosphatase, bilirubin, carbon dioxide, C-reactive protein, hemoglobin, coagulation factors II, VII, and X International Normalized Ratio, potassium, urea, coagulation factors, leukocytes, neutrophils, mean corpuscular hemoglobin concentration, mean cell volume, sodium, platelets, lactate dehydrogenase, neutrophil gelatinase-associated lipocalin, BTP and B2M, soluble urokinase plasminogen activator receptor, cholesterols, triglycerides, blood sugar, glycated hemoglobin, insulin, markers of the effect, and plasma levels of medication. Calculation of eGFR will be based on the Chronic Kidney Disease Epidemiology Collaboration [131–133], Berlin Initiative Study [132], Full Age Spectrum [42, 134, 135], Lund-Malmö revised [136], the Caucasian and Asian pediatric and adult subjects [137], Modification of Diet in Renal Disease [138], and Cockcroft-Gault [42] equations based on creatinine, cystatin C, B2M or BTP, or a combination of the markers.	t_1_, t_2_, t_3_, t_GFR_
Health economics	Use of health care services will be collected from the following registries: National Patient Registry, National Health Insurance Registry, The Danish National Prescription Registry [139], and local databases in the municipalities.	t_1-_ t_3_ and t_1_-t_1y_
Descriptive variables	Participant characteristics are based on participant self-report or obtained from the medical journal and include sex, age, civil status, living conditions, education, smoking status, alcohol consumption, physical activity level, use of home- and health care, early warning score and diagnoses.	t_1_

Timepoint for: baseline: t1, follow-up 8 weeks after discharge: t2, follow-up 16 weeks after discharge: t3, follow-up by telephone 1 year after discharge: t1y, assessment of glomerular filtration rate (GFR): tGFR

### Sample size

The trial has three primary endpoints that build on mutually independent hypotheses [140]. Therefore, sample size calculations were performed for each endpoint as described below. All sample size calculations are performed for a significance level of 5%, power of 80%, and a dropout rate of 25%. Ethical approval was obtained for the inclusion of 200 participants in total to ensure enough power for all primary endpoints.

- 1: Based on a *t* test, a sample size of 120 is required to detect a clinical difference of 3 points (SD=5 points [36]) in MAI score change from baseline to 8 weeks after discharge between the control group and pooled intervention group (MED and MULTI-NUT-MED intervention).
- 2: Based on a paired *t* test, a sample size of 62 participants is required to detect a clinically relevant difference of 4.0 mL/min/1.73m^2^ (SD=15, a R=0.73 [141]) between mGFR and eGFR based on the combination of creatinine and cystatin C. GFR is measured at baseline for all trial participants regardless of group allocation.
- 3: Based on a *t* test, a sample size of 130 participants with or at risk of malnutrition is required to detect a clinically relevant difference of 0.15 points [142] (SD=0.27 [143]) in EuroQol-5D-5L score change from baseline to 16 weeks after discharge between the control group and MULTI-NUT-MED intervention group, assuming equal allocation in the two groups.

Given that patients are included independently of nutrition status and approximately 70% of patients are expected to have or be at risk of malnutrition [4], a total of 185 participants is required to achieve a sample size of 130 participants with or at risk of malnutrition.

## Methods: assignment of interventions

### Allocation sequence generation

Participants are randomly allocated to either the intervention group or control group in blocks of 8 and stratified by age (<75 or ≥75 years of age), sex, and municipality. A random 1:1 sequence of two letters was generated for each block, with the statistical software R 3.6.0 (R Foundation for Statistical Computing, Vienna, Austria) using the sampling function. The randomization sequence was generated by a statistician uninvolved in the trial, and who was unaware of which letter was assigned to which group.

### Allocation concealment mechanisms and implementation

Allocation concealment is achieved as the person performing patient recruitment and data collection (blinded) does not have access to the randomization sequences and is not involved in the randomization. Persons not involved in recruitment will inform intervention staff and participants about group allocation, based upon the randomization sequence. The randomization is performed immediately after a participant has consented to participate and is based solely on stratification-relevant data. The staff performing the interventions (unblinded) are informed immediately after randomization. Participants are informed after baseline data has been collected.

### Blinding

The nature of the intervention does not allow blinding of participants or personnel performing the intervention. However, the outcome assessor is blinded to the result of the randomization throughout the trial, and all data analysis is performed blinded.

## Methods: data collection, management, and analysis

### Data collection methods

Depending on the outcome, data collection is performed at baseline (t_1_), 8 weeks after discharge (t_2_), 16 weeks after discharge (t_3_), 1 year after discharge (t_1y_), and at the GFR-measurement (t_GFR_) (Tables 1 and 2).

Data collection is performed by the same assessor whenever possible to minimize variation. All assessors are provided supervised training in data collection by primary investigator ALA, MBH, RLN, AKB, or HGJL until deemed ready to perform data collection unsupervised. Data are collected in the ED at baseline and in each participants’ home during follow-up. If a participant declines full data collection at follow-up, then they are offered minimal data collection consisting of blood samples, body weight, MNA-SF, EuroQol-5D-5L, blood pressure, heart rate, hand grip strength, and body composition with BIA. If the participant declines minimal data collection, then they are offered to complete any components of data collection that can be performed by telephone interview. A full medication list for each participant is recorded at every follow-up visit, and the patient’s diagnosis codes and laboratory tests are accessed in the patient chart and stored for future MAI and AOU scores assessment. A full medication list for each participant is recorded at every follow-up visit, and the patient’s diagnosis codes and laboratory tests are accessed in the patient chart and stored for future calculation of MAI and AOU scores. A self-reported medication list is obtained by a senior pharmacist through interviewing the participant, and this list is cross-referenced with the Shared Medication Card. If there are any discrepancies between medications reported by the patient and those found in the Shared Medication Card, then the senior pharmacist confirms the medications by contacting the participant’s relatives, home care personnel, or GP. Registry data (see Table 2) are obtained retrospectively.

### Data management

Procedures for data entry, coding, security, and storage have been approved by the Danish Data Protection Agency (VD-2018-390). Whenever possible, data are entered directly into REDCap (Research Electronic Data Capture, Vanderbilt University, Nashville, USA). If data are collected on paper case report forms, then a double entry in REDCap is performed. Data analysis will be performed in SAS Enterprise Guide version 9.4 M5 (SAS Institute Inc., Gary, NC, USA) and R, Version 3.6.1 (R Foundation for Statistical Computing, Vienna, Austria).

### Statistical methods

#### Primary outcomes

Differences in MAI score, change from baseline to 8 weeks after discharge between the control group and pooled intervention group (MED and MULTI-NUT-MED intervention), will be evaluated by analysis of covariance (ANCOVA). The primary analysis will model the randomization group and baseline MAI-score as independent variables by the intention-to-treat principle. Additional analyses will be adjusted for potential confounders that are unequally distributed between groups despite randomization. Missing data on the primary end point will be accounted for by multiple imputations using the PROC MI procedure with fully conditional specification (FCS) and a minimum of 100 imputations. Imputation will use earlier measurements of the imputed outcome, number of medications, and randomization group, along with age and sex for prediction models.

Differences between mGFR and eGFR based on both the combination of creatinine and cystatin C will be evaluated by mixed linear regression analysis.

The difference in EuroQol-5D-5L score change from baseline to 16 weeks after discharge between the control group and MULTI-NUT-MED intervention group will be evaluated by ANCOVA. The primary analysis will model randomization group and baseline EuroQol-5D-5L as independent variables with either intention-to-treat analysis or per-protocol analysis, where participants are considered compliant if they received and were compliant to at least one intervention if one was recommended, two if two were recommended, two if three were recommended, three if four were recommended, and four if five or six were recommended. Additional analyses will be adjusted for potential confounders that are unequally distributed between groups despite randomization. Missing data on the primary endpoint will be accounted for by multiple imputations using the PROC MI procedure with fully conditional specification (FCS) and a minimum of 100 imputations. Imputation will use earlier measurements of the imputed outcome along with age, sex, and diagnosis codes for prediction models.

#### Secondary outcomes

The difference in health care costs between the control group and MULTI-NUT-MED intervention group will be evaluated in two stages. In the first stage, the share of participants having zero costs is compared by *t* test or similar. In the second stage, the magnitude of strictly positive health care costs is compared by generalized linear models [144]. Quality-adjusted life-years (QALYs) will be computed based on survival, EuroQol-5D-5L data collection, and Danish preference weights [145]. Incremental cost-effectiveness ratio (ICER) will be computed by dividing the difference in development in health care costs from inclusion to 52 weeks after inclusion between groups with the difference in change in QALYs between groups. The ICERs will be bootstrapped and reported with confidence limits from the bootstrapping, in scatter plots, and in cost-effectiveness acceptability curves.

The difference in AOU score change from baseline to 8 weeks after discharge between the control group and pooled intervention group (MED or MULTI-NUT-MED intervention) will be evaluated similarly to the difference in MAI score change.

DNA sequencing of fecal bacteria will be mapped to an integrated catalog of reference genes of the human gut microbiome in order to generate an operational taxonomic unit. Microbial species and gene diversity (the gut microbiome composition) will be calculated with the Shannon index. Differences in the Shannon index between the control group and intervention group will be evaluated by ANCOVA adjusted for baseline values. Gut microbiome diversity will be evaluated by descriptive analyses in relation to dietary components and nutritional status evaluated by MNA-SF and Global Leadership Initiative on Malnutrition (GLIM) criteria.

GLIM criteria will be validated against in-depth nutritional assessments performed independently by two blinded and trained nutritional experts (semi-gold standard). The nutritional assessment will include factors related to nutritional status, e.g., dietary intake, factors impeding nutritional intake, appetite, and disease history including LOS and readmissions, body composition, weight history, biochemistry, and physical and psychological wellbeing. The prevalence of malnutrition will be evaluated with descriptive analyses based on specific combinations of phenotypic and etiologic indicators in the GLIM criteria. Finally, statistical agreement analysis between the three malnutrition classifications (MNA-SF, ESPEN, and GLIM) will be evaluated by agreement analysis at admission and up to 16 weeks after discharge. Based on in-depth nutritional assessments, participants only meeting one or two malnutrition classifications will be compared to participants meeting all three malnutrition classifications.

The association between non-GFR determinants and differences between mGFR and eGFR will be evaluated by linear regression analysis. Overall agreement between chronic kidney disease classification based on mGFR and eGFR will be evaluated by Kappa statistics. Results of pharmacogenetic testing including frequency of gene variations and medication interventions based on these variations will be evaluated by descriptive statistics.

Prevalence of frailty will be calculated at baseline and follow-up using three different screening tools (FRAIL, Fried’s frailty phenotype, and FI-OutRef). The effect of the intervention on change in frailty between the baseline and follow-up will be evaluated by mixed models with frailty as the dependent variable and a random effect on participant number, to account for multiple measurements from the same participant. The effect will be measured as an interaction of group and time. Depending on the goodness of fit it may be necessary to categorize frailty. Overall agreement between frailty classification based on the different screening tools will be evaluated by Kappa statistics and mixed models. If the intervention is determined to have a significant effect on frailty, then the agreement between frailty classifications will only be performed using baseline data.

All additional confirmative outcomes will be evaluated by *t* test, Kruskal-Wallis test, chi-square test, two proportions *z* test, or Friedman’s analysis of variance depending on the distribution of the relevant variable. The analysis will be adjusted for multiple testing. Associations between variables will be evaluated by adjusted multiple regression models, and repeated measurements will be evaluated by mixed models of paired *t* tests or Fisher’s exact test. Missing data on secondary endpoints will be accounted for with multiple imputations as previously described.

### Data monitoring, harms, and auditing

The trial is regarded as low-risk and designed by a highly experienced multidisciplinary research team. Interventions are based on well-established recommendations why no adverse events are expected. Therefore, establishing a data monitoring committee was considered unnecessary and no interim analyses will be performed. However, all adverse event will be registered, and appropriate actions will be taken for all participants regardless of group allocation. Participant and persons responsible for data collection are instructed to contact the participant’s GP or study geriatrician if symptoms or conditions arise that may be related to the MED intervention or severely inappropriate medications. Participants are encouraged to raise trial related questions to trial staff and to report all events related to the trial. A physician will evaluate any abnormal blood test results collected for the trial. Participants receiving physiotherapy are monitored for unintentional weight loss. Missing or inaccurate data will be audited daily by trial staff, and the research team will have bimonthly progress meetings to confirm progress and ensure scientific quality.

## Discussion

The trial has several strengths and limitations. The major strength of the trial is its randomized controlled study design. The control group allows identification of possible effects of the intervention that are beyond a patients’ normal capacity to recover, which is known to be high in acutely admitted older patients [146]. Another strength of the trial is that the intervention is performed in the same setting where it is designed to be implemented. A third strength of the trial is the use of ^99m^Tc-DTPA plasma clearance for determining mGFR, which is accepted as the gold standard in clinical settings. However, many equations to estimated GFR are developed using Inulin (plasma or urinary) clearance as a reference. Quality of life has a broad definition, and many tools to measure different aspects of quality of life exist. The strength in using EuroQol-5D-5L is that it comprises both the physical and mental aspects of the quality of life and is widely used in nutrition research and that it can be used in health economic evaluations.

A limitation to the trial design is the lack of double-blinding, which may introduce performance bias. However, double-blinding is not possible due to the nature of the intervention. Another limitation is the discrepancy in staff attention required for the control group and intervention group. Participants receiving the MULTI-NUT-MED intervention, for example, will likely require more interpersonal interaction, which may bias the results of the trial. A third limitation is that several components of the MULTI-NUT-MED intervention are based primarily on self-reported questionnaires, which most likely leads to the underutilization of the intervention.

In conclusion, this trial will provide new information on strategies for optimizing treatment of inappropriate medication prescribing and malnutrition and provide new knowledge regarding the accuracy of eGFR among older medical patients admitted to the ED.

## Supplementary Information

**Additional file 1.** Informed consent. SPIRIT-Guideline checklist.

## Data Availability

The handling of personal information complies with the Danish regulations and EU´s General Data Protection Regulation (GDPR). The project has been listed with the legal office of the Capital Region. ALA, MBH, RLN, LMJ, AKB, HGJ-L, EI, and OA will have access to the final trial dataset. Due to regulations from the Danish Data Protection Act and GDPR, the dataset from our trial is not publicly available as the data contain identifying or sensitive information that could compromise the privacy of the participants. By reasonable request to the corresponding author, the dataset can possibly be made available.

## Abbreviations

ADL: Activities of daily living
AMPS: Assessment of motor and process skills
ANCOVA: Analysis of covariance
AOU: Assessment of underutilization
BIA: Bioelectric impedance analysis
BTP: Beta trace protein
BMI: Body mass index
B2M: Beta-2-microglobulin
DEMMI: De Mortons Mobility Index
DNA: Deoxyribonucleic acid
DXA: Dual X-ray absorptiometry
EAT: Eating Assessment Tool
ED: Emergency department
eGFR: Estimated glomerular filtration rate
ESPEN: European Society of Clinical Nutrition and Metabolism
EuroQol-5D-5L: EuroQol-5 dimensions-5 levels
FCS: Fully conditional specification
FOTT: Facial oral tract therapy
FRS: Functional Recovery Score
GDS: Geriatric Depression Score
GFR: Glomerular filtration rate
GLIM: Global Leadership Initiative on Malnutrition
GP: General practitioner
ICER: Incremental cost-effectiveness ratio
LOS: Length of stay
MAI: Medication Appropriateness Index
MED intervention: Medication review intervention
mGFR: Measured glomerular filtration rate
MNA-SF: Mini Nutritional Assessment–Short Form
MULTI-NUT-MED intervention: Individualized multimodal and transitional nutritional intervention
ONS: Oral nutritional supplements
OTIPM: Occupational Therapy Intervention Process Model
PIM: Potentially inappropriate medication
REDCap: Research electronic data capture
SD: Standard deviation
SPIRIT: Standard Protocol Items: Recommendations for International Trials
VAS: Visual analog scale
QALY: Quality-adjusted life year
^99m^TC-DTPA: ^99m^Technetium–diethylenetriaminepentaacetic acid

## References

[1] Statistikbanken [Internet]. [cited 2016 Oct 21]. Available from: http://www.statistikbanken.dk/statbank5a/default.asp?w=1920.

[2] Wittenberg R, Sharpin L, McCormick B, Hurst J. Understanding emergency hospital admissions of older people. Oxford: Centre for Health Service Economics & Organisation; 2014. p. 86. Report No.: 6

[3] HCUPnet, Healthcare Cost and Utilization Project. HCUPnet-Emergency Department National Statistics [Internet]: HCUPnet; 2016. [cited 2020 Nov 4]. Available from: /

[4] Lawson-Smith L, Petersen J, Jensen PS, Sivertsen DM, Pedersen MM, Ellekilde G (2015). Nutritional risk in acutely admitted older medical patients. Am J Food Nutr.

[5] Pereira GF, Bulik CM, Weaver MA, Holland WC, Platts-Mills TF (2015). Malnutrition among cognitively intact, noncritically ill older adults in the emergency department. Ann Emerg Med..

[6] Bolado Jiménez C, Fernádez Ovalle H, Muñoz Moreno M, de la Fuente RA, de Luis Román D (2019). Undernutrition measured by the Mini Nutritional Assessment (MNA) test and related risk factors in older adults under hospital emergency care. Nutrition.

[7] Burks CE, Jones CW, Braz VA, Swor RA, Richmond NL, Hwang KS, et al. Risk factors for malnutrition among older adults in the emergency department: a multicenter study. J Am Geriatr Soc. 2017;65(8):1741–7. 10.1111/jgs.14862. 10.1111/jgs.14862 PMC5555801 28322438

[8] Griffin A, O’Neill A, O’Connor M, Ryan D, Tierney A, Galvin R (2020). The prevalence of malnutrition and impact on patient outcomes among older adults presenting at an Irish emergency department: a secondary analysis of the OPTI-MEND trial. BMC Geriatr..

[9] Jensen LD, Andersen O, Hallin M, Petersen J (2014). Potentially inappropriate medication related to weakness in older acute medical patients. Int J Clin Pharm..

[10] Damoiseaux-Volman BA, Medlock S, Raven K, Sent D, Romijn JA, van der Velde N, Abu-Hanna A (2021). Potentially inappropriate prescribing in older hospitalized Dutch patients according to the STOPP/START criteria v2: a longitudinal study. Eur J Clin Pharmacol..

[11] Thomas RE, Thomas BC (2019). A Systematic Review of Studies of the STOPP/START 2015 and American Geriatric Society Beers 2015 Criteria in Patients ≥ 65 Years. Curr Aging Sci..

[12] San-José A, Agustí A, Vidal X, Formiga F, López-Soto A, Fernández-Moyano A, García J, Ramírez-Duque N, Torres OH, Barbé J, Potentially Inappropriate Prescription in Older Patients in Spain (PIPOPS) Investigators' Project (2014). Inappropriate prescribing to older patients admitted to hospital: a comparison of different tools of misprescribing and underprescribing. Eur J Intern Med..

[13] Janssen B, Szende A, Szende A, Janssen B, Cabases J (2014). Population Norms for the EQ-5D. Self-reported population health: an international perspective based on EQ-5D [Internet].

[14] Andreasen J, Gobbens RJJ, Eriksen HH, Overvad K (2019). Health-related quality of life at hospital discharge as a predictor for 6-month unplanned readmission and all-cause mortality of acutely admitted older medical patients. Q Life Res..

[15] Rasheed S, Woods RT (2014). An investigation into the association between nutritional status and quality of life in older people admitted to hospital. J Hum Nutr Diet..

[16] Midão L, Giardini A, Menditto E, Kardas P, Costa E (2018). Polypharmacy prevalence among older adults based on the survey of health, ageing and retirement in Europe. Arch Gerontol Geriatr.

[17] Juul-Larsen HG, Christensen LD, Bandholm T, Andersen O, Kallemose T, Jørgensen LM, Petersen J. Patterns of Multimorbidity and Differences in Healthcare Utilization and Complexity Among Acutely Hospitalized Medical Patients - A Latent Class Approach. Clin Epidemiol. 2020.10.2147/CLEP.S226586. 10.2147/CLEP.S226586 PMC7053819 32184671

[18] Vivanti A, Isenring E, Baumann S, Powrie D, O’Neill M, Clark D (2015). Emergency department malnutrition screening and support model improves outcomes in a pilot randomised controlled trial. Emerg Med J..

[19] Christensen J, Fredslund EK (2013). Fælles ældre. Opgørelse af 65+ borgere i hjemmeplejen og i hospitalssektoren [Internet]. KORA. Det Nationale Institut for kommuners og regioners analyse og forskning.

[20] Spinewine A, Schmader KE, Barber N, Hughes C, Lapane KL, Swine C, Hanlon JT (2007). Appropriate prescribing in elderly people: how well can it be measured and optimised?. Lancet..

[21] Frank C (2014). Deprescribing: a new word to guide medication review. CMAJ..

[22] Hilmer SN, McLachlan AJ, Couteur DGL (2007). Clinical pharmacology in the geriatric patient. Fundam Clin Pharmacol.

[23] Kongkaew C, Noyce PR, Ashcroft DM (2008). Hospital admissions associated with adverse drug reactions: a systematic review of prospective observational studies. Ann Pharmacother..

[24] Scott IA, Hilmer SN, Reeve E, Potter K, Le Couteur D, Rigby D (2015). Reducing inappropriate polypharmacy: the process of deprescribing. JAMA Intern Med..

[25] Mangoni AA, Jackson SHD (2004). Age-related changes in pharmacokinetics and pharmacodynamics: basic principles and practical applications. Br J Clin Pharmacol..

[26] Papastergiou J, Tolios P, Li W, Li J (2017). The Innovative Canadian Pharmacogenomic Screening Initiative in Community Pharmacy (ICANPIC) study. Journal of the American Pharmacists Association: JAPhA..

[27] Graabaek T, Kjeldsen LJ (2013). Medication reviews by clinical pharmacists at hospitals lead to improved patient outcomes: a systematic review. Basic Clin Pharmacol Toxicol..

[28] Ravn-Nielsen LV, Duckert M-L, Lund ML, Henriksen JP, Nielsen ML, Eriksen CS (2018). Effect of an in-hospital multifaceted clinical pharmacist intervention on the risk of readmission: a randomized clinical trial. JAMA Intern Med.

[29] Graabaek T, Hedegaard U, Christensen MB, Clemmensen MH, Knudsen T, Aagaard L (2019). Effect of a medicines management model on medication-related readmissions in older patients admitted to a medical acute admission unit-a randomized controlled trial. J Eval Clin Pract..

[30] Christensen M, Lundh A (2013). Medication review in hospitalised patients to reduce morbidity and mortality. Cochrane Database Syst Rev..

[31] Nielsen TRH, Honoré PH, Rasmussen M, Andersen SE (2017). Clinical effects of a pharmacist intervention in acute wards - a randomized controlled trial. Basic Clin Pharmacol Toxicol..

[32] Skjøt-Arkil H, Lundby C, Kjeldsen LJ, Skovgårds DM, Almarsdóttir AB, Kjølhede T, Duedahl TH, Pottegård A, Graabaek T (2018). Multifaceted pharmacist-led interventions in the hospital setting: a systematic review. Basic Clin Pharmacol Toxicol..

[33] Bergkvist A, Midlöv P, Höglund P, Larsson L, Eriksson T (2009). A multi-intervention approach on drug therapy can lead to a more appropriate drug use in the elderly. LIMM-Landskrona Integrated Medicines Management. J Eval Clin Pract..

[34] Spinewine A, Swine C, Dhillon S, Lambert P, Nachega JB, Wilmotte L (2007). Effect of a collaborative approach on the quality of prescribing for geriatric inpatients: a randomized, controlled trial. J Am Geriatr Soc..

[35] Hellström LM, Bondesson A, Höglund P, Midlöv P, Holmdahl L, Rickhag E, Eriksson T (2011). Impact of the Lund Integrated Medicines Management (LIMM) model on medication appropriateness and drug-related hospital revisits. Eur J Clin Pharmacol..

[36] Houlind MB, Andersen AL, Treldal C, Jørgensen LM, Kannegaard PN, Castillo LS, Christensen LD, Tavenier J, Rasmussen LJH,Ankarfeldt MZ, et al. A Collaborative Medication Review Including Deprescribing for Older Patients in an Emergency Department: A LongitudinalFeasibility Study J Clin Med. 2020;9:348. 10.3390/jcm9020348. 10.3390/jcm9020348 PMC7074203 32012721

[37] Zhou S-F, Liu J-P, Chowbay B (2009). Polymorphism of human cytochrome P450 enzymes and its clinical impact. Drug Metab Rev.

[38] Helldén A, Bergman U, von Euler M, Hentschke M, Odar-Cederlöf I, Ohlén G (2009). Adverse drug reactions and impaired renal function in elderly patients admitted to the emergency department: a retrospective study. Drugs Aging..

[39] Wehling M (2013). Drug Therapy for the Elderly [Internet].

[40] Inker LA, Levey AS, Coresh J (2018). Estimated glomerular filtration rate from a panel of filtration markers-hope for increased accuracy beyond measured glomerular filtration rate?. Adv Chronic Kidney Dis..

[41] Foster MC, Levey AS, Inker LA, Shafi T, Fan L, Gudnason V, Katz R, Mitchell GF, Okparavero A, Palsson R, Post WS, Shlipak MG (2017). Non-GFR determinants of low-molecular-weight serum protein filtration markers in the elderly: AGES-Kidney and MESA-Kidney. Am J Kidney Dis..

[42] Pottel H, Schaeffner E, Ebert N (2018). Evaluating the diagnostic value of rescaled β-trace protein in combination with serum creatinine and serum cystatin C in older adults. Clin Chim Acta..

[43] Rasheed S, Woods RT (2013). Malnutrition and quality of life in older people: a systematic review and meta-analysis. Ageing Res Rev.

[44] Marshall S, Bauer J, Isenring E (2014). The consequences of malnutrition following discharge from rehabilitation to the community: a systematic review of current evidence in older adults. J Hum Nutr Diet.

[45] Buurman BM, Hoogerduijn JG, de Haan RJ, Abu-Hanna A, Lagaay AM, Verhaar HJ, et al. Geriatric conditions in acutely hospitalized older patients: prevalence and one-year survival and functional decline. PLoS One. 2011;6(11):e26951. 10.1371/journal.pone.0026951. 10.1371/journal.pone.0026951 PMC3215703 22110598

[46] Sobotka L. Basics in clinical nutrition. 4. ESPEN; (ESPEN Blue Book; vol. 2011). Available from: http://www.espen.org/espen-blue-book.

[47] Carrión S, Cabré M, Monteis R, Roca M, Palomera E, Serra-Prat M, Rofes L, Clavé P (2015). Oropharyngeal dysphagia is a prevalent risk factor for malnutrition in a cohort of older patients admitted with an acute disease to a general hospital. Clin Nutr..

[48] Fávaro-Moreira NC, Krausch-Hofmann S, Matthys C, Vereecken C, Vanhauwaert E, Declercq A, Bekkering GE, Duyck J (2016). Risk factors for malnutrition in older adults: a systematic review of the literature based on longitudinal data. Adv Nutr..

[49] Schilp J, Wijnhoven HAH, Deeg DJH, Visser M. Early Determinants for the Development of Undernutrition in an Older General Population: Longitudinal Aging Study Amsterdam. Br J Nutr. 2011;106:708–17. 10.1017/S0007114511000717. 10.1017/S0007114511000717 21450117

[50] van der Pols-Vijlbrief R, Wijnhoven HAH, Schaap LA, Terwee CB, Visser M (2014). Determinants of protein-energy malnutrition in community-dwelling older adults: a systematic review of observational studies. Ageing Res Rev..

[51] Mowé M, Bøhmer T (2002). Reduced appetite. A predictor for undernutrition in aged people. J Nutr Health Aging..

[52] Sørbye LW, Schroll M, Finne-Soveri H, Jonsson PV, Topinkova E (2008). Unintended weight loss in the elderly living at home: the aged in Home Care Project (AdHOC). J Nutr Health Aging.

[53] Jyrkkä J, Enlund H, Lavikainen P, Sulkava R, Hartikainen S (2011). Association of polypharmacy with nutritional status, functional ability and cognitive capacity over a three-year period in an elderly population. Pharmacoepidemiology and Drug Safety..

[54] Wolff A, Joshi RK, Ekström J, Aframian D, Pedersen AML, Proctor G, Narayana N, Villa A, Sia YW, Aliko A, McGowan R, Kerr AR, Jensen SB, Vissink A, Dawes C (2017). A guide to medications inducing salivary gland dysfunction, xerostomia, and subjective sialorrhea: a systematic review sponsored by the world workshop on oral medicine VI. Drugs R D..

[55] Aung TY, Soo S (2016). Drugs induced nausea and vomiting: an overview. IOSR J Pharm Biol Sci.

[56] Fosnes GS, Lydersen S, Farup PG (2011). Constipation and diarrhoea - common adverse drug reactions? A cross sectional study in the general population. BMC Clin Pharmacol.

[57] Gervasio JM, Boullata JI, Armenti VT (2010). Drug-Induced Changes to Nutritional Status. Handbook of drug-nutrient interactions [Internet].

[58] Volkert D, Beck AM, Cederholm T, Cruz-Jentoft A, Goisser S, Hooper L, Kiesswetter E, Maggio M, Raynaud-Simon A, Sieber CC, Sobotka L, van Asselt D, Wirth R, Bischoff SC (2019). ESPEN guideline on clinical nutrition and hydration in geriatrics. Clin Nutr.

[59] Chan A-W, Tetzlaff JM, Altman DG, Laupacis A, Gøtzsche PC, Krlea-Jerić K (2015). SPIRIT 2013 Statement: defining standard protocol items for clinical trials. Rev Panam Salud Pública.

[60] Rubenstein LZ, Harker JO, Salvà A, Guigoz Y, Vellas B (2001). Screening for undernutrition in geriatric practice: developing the short-form mini-nutritional assessment (MNA-SF). J Gerontol A Biol Sci Med Sci..

[61] VIP-lægelig vurdering og journalføring [Internet]. [cited 2020 Nov 24]. Available from: https://vip.regionh.dk/VIP/Admin/GUI.nsf/Desktop.html?open&openlink=http://vip.regionh.dk/VIP/Slutbruger/Portal.nsf/Main.html?open&unid=XD9D3972FF22CC041C1257F64004A11DF&dbpath=/VIP/Redaktoer/RH.nsf/&windowwidth=1100&windowheight=600&windowtitle=S%F8g.

[62] Shared Medicine Card [Internet]. [cited 2020 Nov 20]. Available from: https://www.danishhealthdata.com/find-health-data/,-w-.

[63] Regionale Ernæringskomitéer i Region Hovedstaden og Region Sjælland. Ernæringsscreening - vurdering og dokumentation hos voksne - vejledning [Internet]. Region Hovedstaden; [cited 2020 Aug 25]. Available from: https://vip.regionh.dk/VIP/Admin/GUI.nsf/Desktop.html.

[64] O’Mahony D, O’Sullivan D, Byrne S, O’Connor MN, Ryan C, Gallagher P (2014). STOPP/START criteria for potentially inappropriate prescribing in older people: version 2. Age Ageing.

[65] Medbase, Ltd in Turku, Finland. Renbase – drug dosing in renal failure. [cited 2016 Oct 23]; Available from:http://www.medbase.fi/en/professionals/renbase.

[66] pro.medicin.dk – information om medicin [Internet]. [cited 2019 Nov 19]. Available from: https://pro.medicin.dk/.

[67] Lægemiddelstyrelsen. Interaktionsdatabasen.dk. [cited 2016 Oct 23]; Available from: http://www.interaktionsdatabasen.dk/.

[68] Lindegaard Pedersen J, Pedersen PU, Damsgaard EM (2017). Nutritional follow-up after discharge prevents readmission to hospital - a randomized clinical trial. J Nutr Health Aging..

[69] Pedersen JL, Pedersen PU, Damsgaard EM (2016). Early nutritional follow-up after discharge prevents deterioration of ADL functions in malnourished, independent, geriatric patients who live alone–A randomized clinical trial. J Nutr Health Aging..

[70] Anbefalinger for den danske institutionskost (2015). Kbh.: Fødevarestyrelsen, Sundhedsstyrelsen, DTU Fødevareinstituttet.

[71] Rofes L, Arreola V, Mukherjee R, Clavé P (2014). Sensitivity and specificity of the Eating Assessment Tool and the Volume-Viscosity Swallow Test for clinical evaluation of oropharyngeal dysphagia. Neurogastroenterol Motil.

[72] Belafsky PC, Mouadeb DA, Rees CJ, Pryor JC, Postma GN, Allen J, Leonard RJ (2008). Validity and reliability of the Eating Assessment Tool (EAT-10). Ann Otol Rhinol Laryngol..

[73] Gibson RS. Principles of Nutritional Assessment: Oxford University Press; 2005. p. 930.

[74] VITAKOST | Moderne kost- og ernæringsberegning [Internet]. [cited 2020 Aug 26]. Available from: https://www.vitakost.dk/da/hjem.

[75] Nordic Council of Ministers NC of M (2012). Nordic nutrition recommendations 2012. Nord Nutr Recomm.

[76] Zuckerman JD, Koval KJ, Aharonoff GB, Skovron ML (2000). A functional recovery score for elderly hip fracture patients: II. Validity and reliability. J Orthop Trauma.

[77] Hansen TS, Jakobsen D (2010). A decision-algorithm defining the rehabilitation approach: ‘Facial oral tract therapy’®. Disabil Rehabil.

[78] Fisher AG, Jones BK. Assesment of motor and process skills volume 1: development, standardization and administration manual. 7th ed: Three Star Press, Inc; 2012.

[79] Fisher AG (1998). Uniting practice and theory in an occupational framework. 1998 Eleanor Clarke Slagle Lecture. Am J Occup Ther..

[80] Jones CJ, Rikli RE, Beam WC (1999). A 30-s chair-stand test as a measure of lower body strength in community-residing older adults. Res Q Exerc Sport..

[81] Sundhedsstyrelsen (2013). Værktøjer til tidlig opsporing af sygdomstegn, nedsat fysisk funktionsniveau og underernæring - sammenfatning af anbefalinger. Kbh.

[82] Abellan van Kan G, Rolland Y, Andrieu S, Bauer J, Beauchet O, Bonnefoy M, Cesari M, Donini LM, Gillette-Guyonnet S, Inzitari M, Nourhashemi F, onder G, Ritz P, Salva A, Visser M, Vellas B (2009). Gait speed at usual pace as a predictor of adverse outcomes in community-dwelling older people an International Academy on Nutrition and Aging (IANA) Task Force. J Nutr Health Aging..

[83] Ratamess AN, Alvar AB, Evetoch KT, Housh TJ, Kibler B, Kraemer WJ (2009). Progression models in resistance training for healthy adults. Med Sci Sports Exerc.

[84] de Vries NM, van Ravensberg CD, Hobbelen JSM, Olde Rikkert MGM, Staal JB, Nijhuis-van der Sanden MWG (2012). Effects of physical exercise therapy on mobility, physical functioning, physical activity and quality of life in community-dwelling older adults with impaired mobility, physical disability and/or multi-morbidity: a meta-analysis. Ageing Res Rev.

[85] Pedersen MM, Petersen J, Beyer N, Larsen HG-J, Jensen PS, Andersen O (2019). A randomized controlled trial of the effect of supervised progressive cross-continuum strength training and protein supplementation in older medical patients: the STAND-Cph trial. Trials..

[86] Signorile JF. Bending the aging curve: the complete exercise guide for older adults: Human Kinetics; 2011. p. 330.

[87] Dondzila CJ, Swartz AM, Keenan KG, Harley AE, Azen R, Strath SJ (2016). Translating exercise interventions to an in-home setting for seniors: preliminary impact on physical activity and function. Aging Clin Exp Res.

[88] Rinaldi P, Mecocci P, Benedetti C, Ercolani S, Bregnocchi M, Menculini G, Catani M, Senin U, Cherubini A (2003). Validation of the five-item geriatric depression scale in elderly subjects in three different settings. J Am Geriatr Soc..

[89] Marc LG, Raue PJ, Bruce ML (2008). Screening performance of the 15-item geriatric depression scale in a diverse elderly home care population. Am J Geriatr Psychiatry.

[90] Thomson WM, van der Putten G-J, de Baat C, Ikebe K, Matsuda K, Enoki K, Hopcraft MS, Ling GY (2011). Shortening the xerostomia inventory. Oral Surg Oral Med Oral Pathol Oral Radiol Endodontol.

[91] The EuroQol Group (1990). EuroQol - a new facility for the measurement of health-related quality of life. Health Policy..

[92] Herdman M, Gudex C, Lloyd A, Janssen M, Kind P, Parkin D, Bonsel G, Badia X (2011). Development and preliminary testing of the new five-level version of EQ-5D (EQ-5D-5L). Qual Life Res..

[93] Hanlon JT, Schmader KE, Samsa GP, Weinberger M, Uttech KM, Lewis IK, et al. Method for assessing drug therapy appropriateness. J Clin Epidemiol. 1992;45(10):1045–51. 10.1016/0895-4356(92)90144-C. 10.1016/0895-4356(92)90144-c 1474400

[94] Gallagher PF, O’Connor MN, O’Mahony D (2011). Prevention of potentially inappropriate prescribing for elderly patients: a randomized controlled trial using STOPP/START criteria. Clin Pharmacol Ther..

[95] Samsa GP, Hanlon JT, Schmader KE, Weinberger M, Clipp EC, Uttech KM, Lewis IK, Landsman PB, Cohen HJ (1994). A summated score for the medication appropriateness index: development and assessment of clinimetric properties including content validity. J Clin Epidemiol.

[96] Fleming JS, Zivanovic MA, Blake GM, Burniston M, Cosgriff PS, British Nuclear Medicine Society (2004). Guidelines for the measurement of glomerular filtration rate using plasma sampling. Nucl Med Commun..

[97] Andersen TB, Jødal L, Nielsen NS, Petersen LJ (2019). Comparison of simultaneous plasma clearance of 99mTc-DTPA and 51Cr-EDTA: can one tracer replace the other?. Scand J Clin Lab Invest..

[98] Schmader KE, Hanlon JT, Pieper CF, Sloane R, Ruby CM, Twersky J, Francis SD, Branch LG, Lindblad CI, Artz M, Weinberger M, Feussner JR, Cohen HJ (2004). Effect of an interdisciplinary team on suboptimal prescribing in a long term care facility. Am J Med..

[99] GeneYouIn. Pillcheck. [cited 2016 Oct 23]; Available from: https://www.pillcheck.ca/.

[100] PharmGKB [Internet]. PharmGKB. [cited 2020 Nov 24]. Available from: https://www.pharmgkb.org/.

[101] Clinical Pharmacogenetics Implementation Consortium [Internet]. [cited 2020 Nov 24]. Available from: https://cpicpgx.org/.

[102] DTU Fødevareinstituttet, Det nationale forskningscenter for velfærd. Danskernes Kost og Fysisk aktivitet - billedserie. DTU Fødevareinstituttet. 2011.

[103] Roberts HC, Denison HJ, Martin HJ, Patel HP, Syddall H, Cooper C, Sayer AA (2011). A review of the measurement of grip strength in clinical and epidemiological studies: towards a standardised approach. Age Ageing..

[104] Bodilsen AC, Pedersen MM, Petersen J, Beyer N, Andersen O, Smith LL, Kehlet H, Bandholm T (2013). Acute hospitalization of the older patient: changes in muscle strength and functional performance during hospitalization and 30 days after discharge. Am J Physical Med Rehabil.

[105] Guralnik JM, Simonsick EM, Ferrucci L, Glynn RJ, Berkman LF, Blazer DG, Scherr PA, Wallace RB (1994). A short physical performance battery assessing lower extremity function: association with self-reported disability and prediction of mortality and nursing home admission. J Gerontol.

[106] de Morton NA, Davidson M, Keating JL (2010). Validity, responsiveness and the minimal clinically important difference for the de Morton Mobility Index (DEMMI) in an older acute medical population. BMC Geriatr..

[107] de Morton NA, Meyer C, Moore KJ, Dow B, Jones C, Hill K (2011). Validation of the de Morton Mobility Index (DEMMI) with older community care recipients. Australas J Ageing.

[108] Davenport SJ, de Morton NA (2011). Clinimetric properties of the de morton mobility index in healthy, community-dwelling older adults. Arch Phys Med Rehabil.

[109] de Morton NA, Brusco NK, Wood L, Lawler K, Taylor NF (2011). The de Morton Mobility Index (DEMMI) provides a valid method for measuring and monitoring the mobility of patients making the transition from hospital to the community: an observational study. J Physiother.

[110] ActivPal (TM) [Internet]. [cited 2018 Apr 17]. Available from: http://www.palt.com/products/.

[111] Topp CW, Østergaard SD, Søndergaard S, Bech P (2015). The WHO-5 well-being index: a systematic review of the literature. Psychother Psychosom.

[112] Fried LP, Tangen CM, Walston J, Newman AB, Hirsch C, Gottdiener J, Seeman T, Tracy R, Kop WJ, Burke G, McBurnie MA (2001). Frailty in older adultsevidence for a phenotype. J Gerontol A Biol Sci Med Sci..

[113] Morley JE, Vellas B (2013). Abellan van Kan G, Anker SD, Bauer JM, Bernabei R, et al. Frailty consensus: a call to action. J Am Med Dir Assoc.

[114] Klausen HH, Petersen J, Bandholm T, Juul-Larsen HG, Tavenier J, Eugen-Olsen J (2017). Association between routine laboratory tests and long-term mortality among acutely admitted older medical patients: a cohort study. BMC Geriatr..

[115] Lee SY, Gallagher D (2008). Assessment methods in human body composition. Curr Opin Clin Nutr Metab Care..

[116] InBody [Internet]. [cited 2020 Oct 9]. Available from: http://www.inbody.com/global/product/inbodys10.aspx.

[117] Dødsårsagsregisteret (DAR) - Sundhedsdatastyrelsen [Internet]. [cited 2020 Nov 20]. Available from: https://sundhedsdatastyrelsen.dk/dar.

[118] Bowie CR, Harvey PD (2006). Administration and interpretation of the Trail Making Test. Nat Protoc..

[119] Lezak MD, Howieson DB, Loring DW, Hannay HJ, Fischer JS (2004). Neuropsychological Assessment. 4th Edition. Oxford.

[120] Shapiro AM, Benedict RH, Schretlen D, Brandt J (1999). Construct and concurrent validity of the Hopkins Verbal Learning Test-revised. Clin Neuropsychol..

[121] Folstein MF, Folstein SE, McHugh PR (1975). “Mini-mental state”. A practical method for grading the cognitive state of patients for the clinician. J Psychiatr Res..

[122] Goring H, Baldwin R, Marriott A, Pratt H, Roberts C (2004). Validation of short screening tests for depression and cognitive impairment in older medically ill inpatients. Int J Geriatr Psychiatry..

[123] Kondrup J, Rasmussen HH, Hamberg O, Stanga Z (2003). Ad Hoc ESPEN Working Group. Nutritional risk screening (NRS 2002): a new method based on an analysis of controlled clinical trials. Clin Nutr.

[124] Beck AM, Beermann T, Kjær S, Rasmussen HH (2013). Ability of different screening tools to predict positive effect on nutritional intervention among the elderly in primary health care. Nutrition..

[125] Nordén J, Grönberg AM, Bosaeus I, Forslund HB, Hulthén L, Rothenberg E, Karlsson J, Wallengren O, Slinde F (2015). Nutrition impact symptoms and body composition in patients with COPD. Eur J Clin Nutr..

[126] Wilson M-MG, Thomas DR, Rubenstein LZ, Chibnall JT, Anderson S, Baxi A, Diebold MR, Morley JE (2005). Appetite assessment: simple appetite questionnaire predicts weight loss in community-dwelling adults and nursing home residents. Am J Clin Nutr.

[127] Cederholm T, Jensen GL, Correia MITD, Gonzalez MC, Fukushima R, Higashiguchi T, Baptista G, Barazzoni R, Blaauw R, Coats A, Crivelli A, Evans DC, Gramlich L, Fuchs-Tarlovsky V, Keller H, Llido L, Malone A, Mogensen KM, Morley JE, Muscaritoli M, Nyulasi I, Pirlich M, Pisprasert V, de van der Schueren MAE, Siltharm S, Singer P, Tappenden K, Velasco N, Waitzberg D, Yamwong P, Yu J, van Gossum A, Compher C, Jensen GL, Charlene C, Cederholm T, van Gossum A, Correia MITD, Gonzalez MC, Fukushima R, Higashiguchi T, Baptista G, Barazzoni R, Blaauw R, Coats A, Crivelli A, Evans DC, Gramlich L, Fuchs V, Keller H, Llido L, Malone A, Mogensen KM, Morley JE, Muscaritoli M, Nyulasi I, Pirlich M, Pisprasert V, de van der Schueren MAE, Siltharm S, Singer P, Tappenden K, Velasco N, Waitzberg D, Yamwong P, Yu J (2019). GLIM criteria for the diagnosis of malnutrition - a consensus report from the global clinical nutrition community. Clin Nutr..

[128] Cederholm T, Bosaeus I, Barazzoni R, Bauer J, Van Gossum A, Klek S (2015). Diagnostic criteria for malnutrition – an ESPEN Consensus Statement. Clin Nutr.

[129] EasySampler kits med grundig instruktion. Forskellig version og pakning [Internet]. GP Medical Devices. [cited 2020 Oct 9]. Available from: https://gpmd.dk/easysampler-kits/.

[130] Lewis SJ, Heaton KW (1997). Stool form scale as a useful guide to intestinal transit time. Scandinavian Journal of Gastroenterology..

[131] Levey AS, Stevens LA, Schmid CH, Zhang YL, Castro AF, Feldman HI (2009). A new equation to estimate glomerular filtration rate. Ann Intern Med..

[132] Inker LA, Tighiouart H, Coresh J, Foster MC, Anderson AH, Beck GJ, Contreras G, Greene T, Karger AB, Kusek JW, Lash J, Lewis J, Schelling JR, Navaneethan SD, Sondheimer J, Shafi T, Levey AS (2016). GFR estimation using β-trace protein and β2-microglobulin in CKD. Am J Kidney Dis..

[133] Inker LA, Schmid CH, Tighiouart H, Eckfeldt JH, Feldman HI, Greene T, Kusek JW, Manzi J, van Lente F, Zhang YL, Coresh J, Levey AS, CKD-EPI Investigators (2012). Estimating glomerular filtration rate from serum creatinine and cystatin C. N Engl J Med..

[134] Pottel H, Delanaye P, Schaeffner E, Dubourg L, Eriksen BO, Melsom T (2017). Estimating glomerular filtration rate for the full age spectrum from serum creatinine and cystatin C. Nephrol Dial Transplant.

[135] Pottel H, Hoste L, Dubourg L, Ebert N, Schaeffner E, Eriksen BO, Melsom T, Lamb EJ, Rule AD, Turner ST, Glassock RJ, de Souza V, Selistre L, Mariat C, Martens F, Delanaye P (2016). An estimated glomerular filtration rate equation for the full age spectrum. Nephrol Dial Transplant..

[136] Björk J, Grubb A, Sterner G, Nyman U (2011). Revised equations for estimating glomerular filtration rate based on the Lund-Malmö Study cohort. Scand J Clin Lab Invest..

[137] Grubb A, Horio M, Hansson L-O, Björk J, Nyman U, Flodin M, Larsson A, Bökenkamp A, Yasuda Y, Blufpand H, Lindström V, Zegers I, Althaus H, Blirup-Jensen S, Itoh Y, Sjöström P, Nordin G, Christensson A, Klima H, Sunde K, Hjort-Christensen P, Armbruster D, Ferrero C (2014). Generation of a new cystatin C-based estimating equation for glomerular filtration rate by use of 7 assays standardized to the international calibrator. Clin Chem..

[138] Levey AS, Bosch JP, Lewis JB, Greene T, Rogers N, Roth D (1999). A more accurate method to estimate glomerular filtration rate from serum creatinine: a new prediction equation. Modification of Diet in Renal Disease Study Group. Ann Intern Med..

[139] Kildemoes HW, Sørensen HT, Hallas J (2011). The Danish National Prescription Registry. Scand J Public Health.

[140] Veazie PJ (2006). When to combine hypotheses and adjust for multiple tests. Health Serv Res.

[141] Segarra A, de la Torre J, Ramos N, Quiroz A, Garjau M, Torres I, Azancot MA, López M, Sobrado A (2011). Assessing glomerular filtration rate in hospitalized patients: a comparison between CKD-EPI and four cystatin C-based equations. Clin J Am Soc Nephrol..

[142] Walters SJ, Brazier JE (2005). Comparison of the minimally important difference for two health state utility measures: EQ-5D and SF-6D. Qual Life Res..

[143] Beck AM, Christensen AG, Hansen BS, Damsbo-Svendsen S, Møller TKS (2016). Multidisciplinary nutritional support for undernutrition in nursing home and home-care: A cluster randomized controlled trial. Nutrition..

[144] Briggs A, Claxton K, Sculpher MJ (2006). Decision modelling for health economic evaluation.

[145] Pedersen KM (2003). Værdisætning af sundhed: teorien om kvalitetsjusterede leveår og en dansk anvendelse /.

[146] Van Ancum JM, Scheerman K, Jonkman NH, Smeenk HE, Kruizinga RC, Meskers CGM (2017). Change in muscle strength and muscle mass in older hospitalized patients: A systematic review and meta-analysis. Exp Gerontol..

